# Towards Small Scale: Overview and Applications of Microfluidics in Biotechnology

**DOI:** 10.1007/s12033-022-00626-6

**Published:** 2022-12-14

**Authors:** Anton Enders, Alexander Grünberger, Janina Bahnemann

**Affiliations:** 1 Institute of Technical Chemistry, Leibniz University Hannover, Callinstraße 5, 30167 Hannover, Germany; 2 Institute of Process Engineering in Life Sciences: Microsystems in Bioprocess Engineering, Karlsruhe Institute of Technology, Fritz-Haber-Weg 2, 76131 Karlsruhe, Germany; 3 Institute of Physics, University of Augsburg, Universitätsstraße 1, 86159 Augsburg, Germany

**Keywords:** Industrial biotechnology, Medical biotechnology, Microfluidics, Point-of-use, Point-of-care, Organ-on-a-chip, Lab-on-a-chip, Nanofluidics, Microfluidic screening, Biochemical engineering

## Abstract

Thanks to recent and continuing technological innovations, modern microfluidic systems are increasingly offering researchers working across all fields of biotechnology exciting new possibilities (especially with respect to facilitating high throughput analysis, portability, and parallelization). The advantages offered by microfluidic devices—namely, the substantially lowered chemical and sample consumption they require, the increased energy and mass transfer they offer, and their comparatively small size—can potentially be leveraged in every sub-field of biotechnology. However, to date, most of the reported devices have been deployed in furtherance of healthcare, pharmaceutical, and/or industrial applications. In this review, we consider examples of microfluidic and miniaturized systems across biotechnology sub-fields. In this context, we point out the advantages of microfluidics for various applications and highlight the common features of devices and the potential for transferability to other application areas. This will provide incentives for increased collaboration between researchers from different disciplines in the field of biotechnology.

## Introduction

**Fig. 1:**
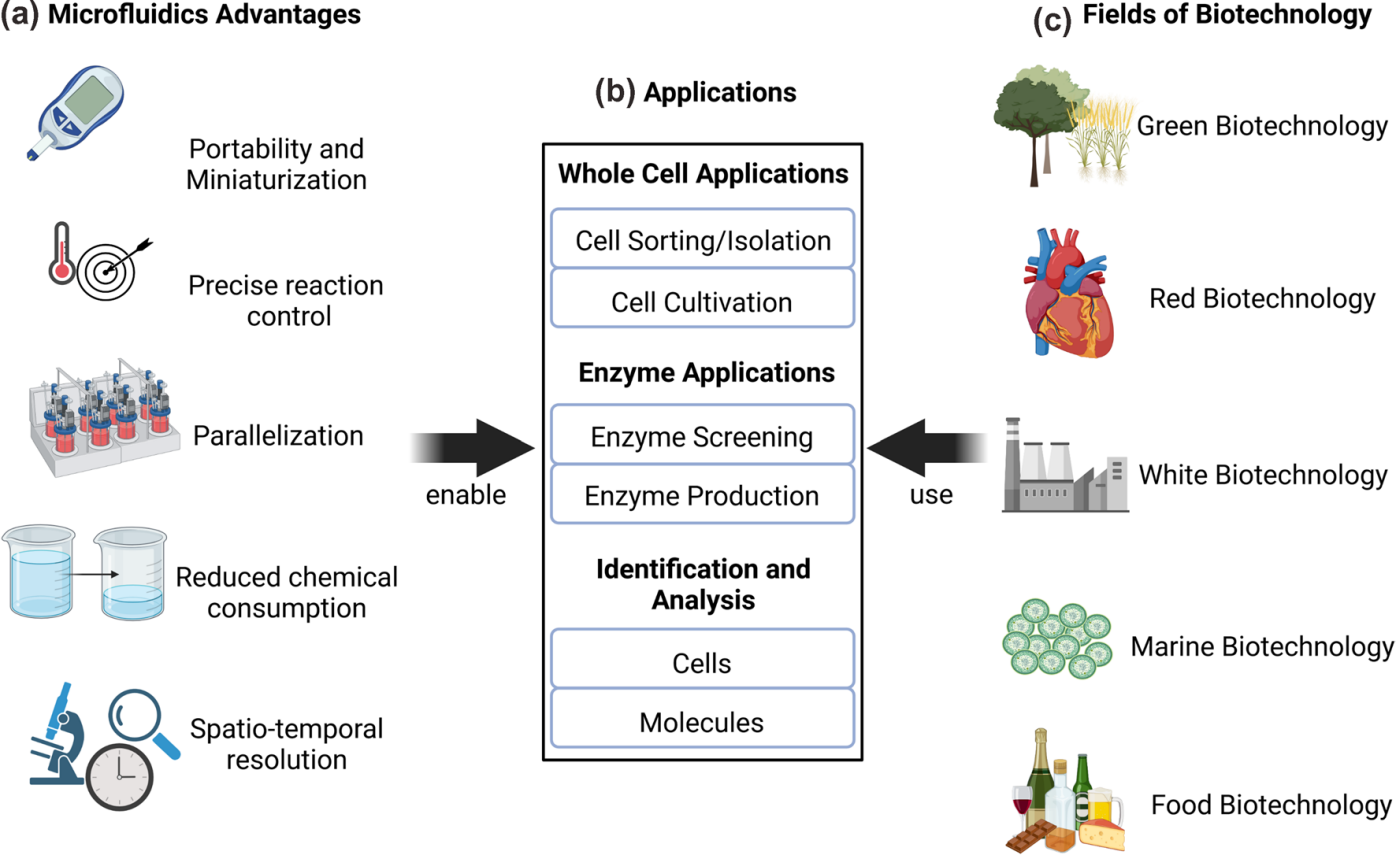
Microfluidic systems enable various applications which are used across all sub-fields of biotechnology (This image was created with BioRender.com)

The wide-ranging discipline of biotechnology covers a tremendous amount of technological ground—from the use of cells for production of pharmaceutical compounds and food aromas, to the development of assays for detection of disease, and even extending to the use of purified enzymes in various industrial applications. In order to clarify which of these many sub-fields of biotechnology are being discussed in a given article, the literature has adopted a useful “color”-based categorization approach (Fig. 1) [1]. “Red” biotechnology involves pharmaceutical and health applications; “yellow” biotechnology covers food science applications; “green” biotechnology encompasses agricultural uses; “blue” biotechnology revolves around marine and fresh water applications; and “white” biotechnology is reserved for those use cases which are purely industrial in nature [1, 2]. (There are additional categories for even more highly specialized biotechnological fields, such as bioinformatics or bioweapons [2], but they are not considered here because they fall outside the scope of this review paper.)

These different biotechnological sub-fields often rely on very similar tools and practices in order to achieve their research and/or production goals. For example, genetic sequencing techniques are crucial for genomic research across all areas of biotechnology—regardless of whether the subject of study is microalgae, animals, plants, or bacteria [3–6]. Having said that, the differences that characterize these sub-fields frequently impact the specific demands that researchers place on the common equipment and procedures in question. Some fields, like marine and food sciences, profit tremendously from the deployment of mobile analytic devices [7–9]. Similarly, point-of-care systems aim to improve diagnostics in healthcare [10, 11]. Other fields require expensive chemicals or samples, meaning that any reduction in the consumption of these materials can substantially cut research costs [12]. Generally speaking, however, researchers across all sub-field do share some common goals: a desire to increase automatization and reliability while simultaneously generating more detailed analytical results. Additionally, parallelization also offers substantial benefits (especially in the form of increased throughput and improved speed) across nearly all areas of biotechnology research and development. It should come as no surprise that new possibilities offered by the increasing adoption and evolution of microfluidic systems have accordingly caught the eyes of researchers working in all biotechnology sub-fields.

A vast number of microfluidic systems suitable for use in biotechnology have already been explored in the literature. The advantages of such miniaturized systems are manifold: The small size of these systems makes portable applications possible, while simultaneously reducing the chemical and sample consumption size required; reaction control is increased due to comparatively fast heat and mass transfer, even as smaller channels and reaction chambers enable far more detailed analysis (e.g., via microscopy); and opportunities for pursuing greater degrees of both automation and parallelization stand to benefit applications in industrial production and research settings alike (see Fig. 1). In this review, we highlight representative examples of how microfluidic systems have been successfully implemented across all of the different sub-fields of biotechnology noted above, while also assessing the relative strengths and perspectives of different microfluidic approaches in each one of these areas. Hereby, we hope to broaden the view of the researchers in their respective fields, identify similar applications in different sub-fields of biotechnology and incentivize increased cooperation across all biotechnology.

## Applications of Microfluidic Systems in Biotechnology

In this chapter, we consider the three primary application types that characterize biotechnology research—whole cell applications, enzyme applications, and identification and analysis—and highlight some specific microfluidic systems that have been successfully deployed in each case.

### Whole Cell Applications

#### Cell Sorting and Cell Isolation

Isolating specific cells from a complex sample and identifying key properties within an individual cell are two important goals that are common to many biotechnological cell sorting and/or screening applications. Cells with higher productivity, certain size and/or optical properties, or other special characteristics can often be identified using a sorting and isolation process—and because all areas of biotechnology employ such processes, many microfluidic devices have been developed with precisely these applications in mind.

**Fig. 2:**
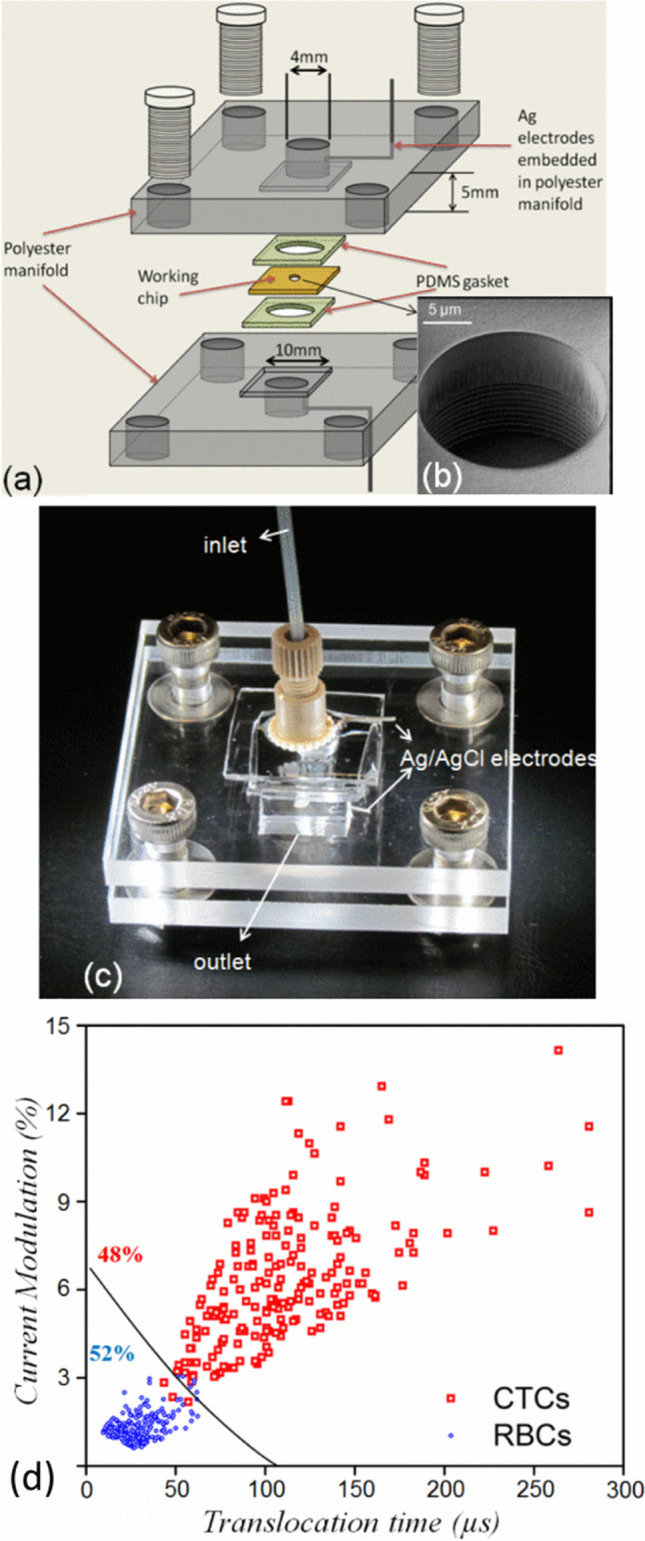
A microfluidic system used for identification of circulating tumor cells (CTCs) and red blood cells (RBCs). **a** Schematic of the microfluidic system with embedded Ag/AgCl electrodes. **b** Detailed view of the separation area in the working chip. **c** The completed microfluidic system. **d** The results of the separation of CTCs and RBCs [14]

There are a number of different methods that can be deployed to separate a target cell from the sample in which it is contained. Dielectrophoresis is one particularly popular approach that is frequently used to separate cells based on differences in electrical properties, since it is a label-free method that does not require any complex and laborious pre-processing or label removing steps [13]. Guo et al*.* have reported success using this technology to develop a small microfluidic impedance cytometer to detect and enumerate cancer cells from red blood cells (see Fig. 2). When paired with computer assisted automatic analysis of the results, the precision of their microfluidic device was comparable to conventional flow cytometers—which are bulky benchtop machines that *do* require the fluorescent labelling of cells [14]. Similarly, Do et al*.* developed a microfluidic enrichment platform for tumor cell detection that used dielectrophoresis to guide tumor cells to the center of the microfluidic system, binding the target cells with antibodies and using a built-in capacitance sensor to identify the cell type [15]. The interaction behavior of cells at different frequency settings was also used by Deng et al*.* to sort microalgae with different lipid contents for biofuel production [16, 17]. Cell sorting and detection is critical for pathogen detection in the context of food production and biomedical production, and while centrifugation and subsequent culturing steps have traditionally been used to detect any undesired bacteria or other organisms, Cheng et al*.* fabricated an integrated dielectrophoretic chip that was successfully deployed to continuously sort and trap *Escherichia coli*, *Lactobacillus* and *Candida albicans* [18].

Another separation technique relies on fluidic manipulation. Instead of inducing a magnetic or electric field, fluid flow is influenced, using special channel geometry and/or varying flows of liquids or gases, to induce desired movement within a group of cells (e.g., to separate cells by size). One popular application of this approach is for the handling of human blood. The detection of bacteria in blood is a critical step in the diagnosis of blood sepsis. Using additional sheath flows, bacteria have been separated from human blood cells in several microfluidic systems [19, 20]. Another strategy is to design special curved channels (like spirals) where cells are focused into certain positions within the channel. These channels can then be used to separate the smaller bacterial cells from the larger blood cells [21]. This separation phenomenon can also be used to separate white blood cells from red blood cells—an important sample preparation step in many diagnostic applications [22]. Similarly, in a protein production process using Chinese Hamster Ovary (CHO) cells, Kwon et al*.* utilized a spiral separation device to separate and remove nonviable cells and cell debris from the bioreactor via a spiral separation device [23]. Spiral separation devices have also been used for cell retention for bioreactors [24]. A spiral microchannel was successfully used in the sub-field of marine biotechnology to sort lipid rich microalgae from invasive diatom [25]. Beebe’s group developed a microfluidic system to sort the sperm and eggs of livestock via controlled liquid and gas flows, with the goal of sequencing the genomes of cattle, poultry, pig and sheep (which allows breeders to more precisely select for desirable traits within their breeding stock [8, 26]).

As demonstrated, microfluidic systems for cell separation and screening have great potential in many fields of biotechnology. While the examples shown here were in a demonstration phase, the unique capabilities of the systems like label-free detection or easy separation of bacteria from blood show great promise for these devices to be deployed in small systems with automated pumps.

#### Cell Cultivation

Cell cultivation is a critical component of many aspects of biotechnological research: both to produce cells, proteins, or other cellular products directly, and also to analyze and better understand cell behavior. Cell cultivation protocols often depend on the natural environment where the specific cell type comes from. For instance, cells from animal tissues often require a surface or matrix to which they can adhere (mimicking the extracellular matrices that exist organically within an animal), while microalgae, yeasts, and bacteria can typically be grown suspended in fluid media.

In white biotechnology, large bioreactors containing thousands of liters of fluid are frequently used for product production, with suspension cells being a prerequisite for cost effectivity. For cultivation analysis, smaller scale cultivations are used to decrease space and media consumption and to facilitate parallelization. In these smaller cultivations, shake flasks or even small well plates can be used for production volumes down to the microliter scale. For adherent cells, however, special culturing flasks with large volumes are typically used, as well as gel matrices (like hydrogels). Microfluidic systems aim to decrease the volume requirements and increase parallelization while simultaneously also enabling researchers to fine-tune environmental conditions and better observe singular cells over a set period of time.

In red biotechnology, a whole field of research has emerged in recent years from the idea of cultivating cells within a microfluidic system: the so-called “organ-on-a-chip”. The aim of organ-on-a-chip systems is to mimic organs of the human body in order to better study the effects of toxins and/or pharmaceuticals on cells within these artificial organs, as well as promote advances in tissue cultivation and facilitate greater understanding of organic organ physiology. These systems often consist of three-dimensional microchannels that are lined with living human cells, which replicate tissue-to-tissue interfaces as well as organ-specific mechanical and biochemical microenvironments [27]. Different compounds can then be injected into this “living” media stream—which is intended to mimic the blood stream that exists within an organism—and cellular behavior can be observed and evaluated. This process allows researchers to test for potential toxicities more efficiently and more rapidly evaluate pharmaceutical candidates in pre-clinical testing situations. Similar principles of chip design can also be applied to other areas of biotechnology: for example, in green biotechnology, the analog system is called “roots-on-a-chip” or “plant-on-a-chip”, and allows researchers to study the interactions of plant root cells and various bacteria or fungi, as well as the reaction of roots to drought environments and/or chemical treatments [28]. In the field of marine technology, microfluidic systems with similar goals have also been used to study not only microalgae, but also fish embryos [9].

Another increasingly popular area of microfluidic research is single cell cultivation and analysis. Cells can show significant heterogeneity within their physiology—and, as a result, bulk measurements of growth and production can only ever reflect the mean value of an aggregate cell population. Investigation undertaken on the single cell level is generally considered to be much more accurate and informative [29], but isolating cells for single cell cultures can be challenging. This challenge can be met through several different means. One option is to generate miniscule droplets of the cell broth in an immiscible fluid (like oil) using droplet microfluidics, and thereby stochastically isolate a single cell within these droplets [30]. This technique has already been successfully used with bacteria, yeast, and other suspension cell lines [29]. Adapting droplet microfluidics to include alginate hydrogels can also enable the cultivation of adherent cells which are suitable for culturing tissues, cancer cells, and stem cells [31–34]. Another technique is the use of microwells, where cells are loaded onto a material with a number of tiny wells (ranging from tens to hundreds of µm big) and the wells are sized such that only individual cells can slot into them. Cells that are not firmly settled into these wells are then washed away [35]. One challenge posed by microwell technology is the need to provide sufficient area for cell growth over the cultivation period, however [36]. Yet another technique is the use of cell traps, whereby single cells are captured from a cell suspension stream. These traps can be based on either a mechanical obstruction [37] or hydrodynamic interactions [38].

**Fig. 3:**
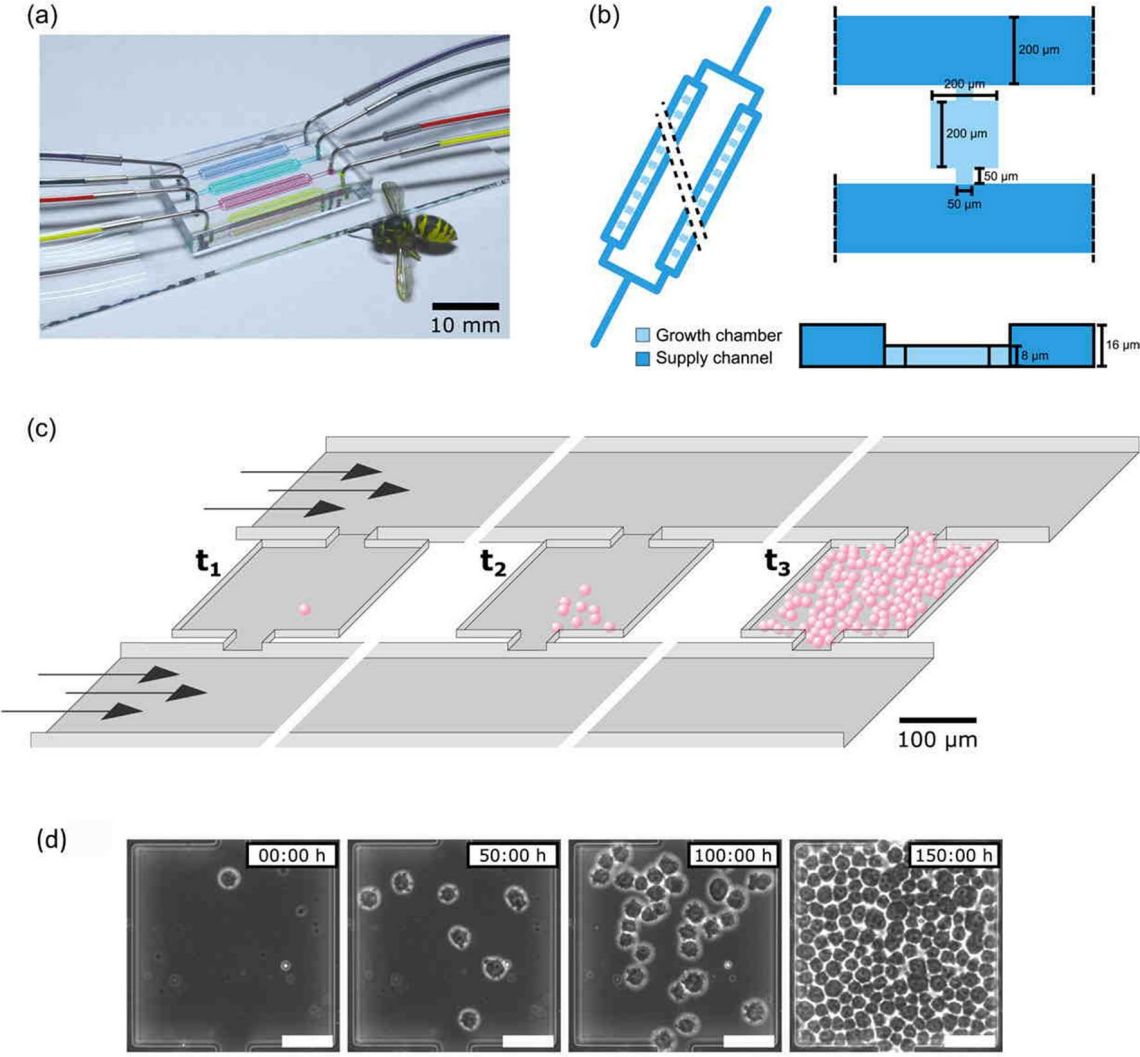
Microfluidic system for single cell cultivation and observation. **a** Microfluidic chip with tube connections for medium perfusion. **b** Schematic of the cultivation chamber setup with multiple growth chambers connected to media supply channels. **c** Schematic overview of the experiments with cells growing over time from a single cell. **d** Microscopic images of CHO-K1 cells at different times during the cultivation [40]. Reprinted with permission from John Wiley and Sons

Parallelization is another key benefit offered by microfluidic systems. The use of cell cultivation parallelization for optimizing cultivation parameters is particularly useful in the context of white and blue biotechnology applications. To facilitate parallelization, microfluidic systems can incorporate several cultivation chambers with fluid input and output channels. Gómez-Sjöberg et al*.* have developed a system with 96 independent culture chambers, each of which can be individually addressed for changes in seeding density, culture medium composition, and feeding schedule. Furthermore, these individual culture chambers also have a relatively small volume of just 60 nL, thus substantially decreasing both media and chemical consumption [39]. Leveraging this principle, Schmitz et al*.* have demonstrated a microfluidic system for parallel cultivation from single cells—opening up the possibility of microscopic observation during media perfusion over several days (see Fig. 3) [40]. Another microfluidic system utilizing parallelization techniques for antimicrobial susceptibility testing was presented by Heuer et al., which lowered the testing time to just 90 minutes, while current clinical practices require more than 8 hours [41].

Using microfluidics for cell cultivation might very well be standard practice in the future. Especially organ-on-a-chip devices will be crucial for pharmaceutical developments by providing higher quality data on efficacy and safety without the need to harm and kill animals [42]. At the same time, advances in organ-on-a-chip technology might also improve cell cultivation in ever smaller vessels in white biotechnology, since the analytical capabilities need to be miniaturized as well for better understanding of cell cultivations.

### Enzyme Applications

Biotechnology encompasses the technological use not only of cells themselves, but also their products (especially, but not exclusively, enzymes). Enzymes are of great importance for the catalyzation of biochemical reactions in the food industry, in the pharmaceutical industry, and even in the production of fine chemicals. Indeed, it is hard to overstate just how critical and ubiquitous enzymes are in many commercial applications—without enzymes, we would not have detergents for washing machines and dishwashers, to cite just one very mundane example. And much like cell applications, many enzyme applications also stand to benefit tremendously from the advantages offered by microfluidic technology.

#### Enzyme Screening

Natural product screening processes that are aimed at identifying new enzymes benefit greatly from lowered reagent consumption rates and relatively high throughput. Ochoa et al*.* have created a droplet microfluidic screening system to detect enzyme inhibitors in natural extracts by attaching a high-performance liquid chromatography (HPLC) system to the droplet microfluidic system, and thereby generating individual liquid droplets that contain an enzyme (*Clostridium perfringens* neuroamidase), a fluorogenic reporter substrate, and individual fractions of the extract of *Pelargonium sidoides* plant roots. This creative use of droplet microfluidics enabled them to substantially reduce their sample volume and achieve a remarkable 50-fold decrease in both enzyme and substrate volume [43].

Similarly, the enhancement of enzyme activity via alteration of amino acid sequence (a process called “directed evolution”) also benefits from the comparatively high throughput that can be achieved through droplet microfluidics. Kintses et al. [44] have developed a microfluidic system capable of creating 3.6 *million* droplets per hour, each containing a single *E. coli* cell. These cells can then be induced to express the protein of interest and lysed inside of the droplet, exposing the protein for later analysis together with plasmid DNA recovery genome sequence analysis in each droplet. The quality of the resulting assay was comparable to that achieved via microplate screening systems—even though the volume of material required to conduct this experiment was exponentially reduced when compared with more traditional methods. Diefenbach et al*.* have developed a similar directed evolution setup, but instead of using the droplet microfluidics for assay analysis and DNA recovers, a mass spectrometer was instead attached to the system—creating a label-free high throughput screening platform [45]. By offering researchers the tantalizing possibility of creating large populations featuring tremendous genetic variations, droplet microfluidics stands poised to become a key technology in future research involving directed evolution [46].

#### Enzyme Production

While enzyme discovery and evolution are important components in the process of enzyme research, the ultimate goal of such research is, of course, to actually utilize enzymes for biocatalysis within chemical reactions. Traditionally, large stainless-steel reactors have been used for biocatalytic reactions; however, miniaturized continuous-flow reactors now allow experiments to be performed with much smaller volumes, thereby offering a significant decrease in the (often-substantial) costs associated with procuring expensive substrate and/or enzymes. Additionally, the parameters of a given reaction can be managed much more easily in continuous-flow reactors, since no mechanical mixing is required and reactions can therefore be accelerated via enhanced mass transfer and decreased reaction time [47]. The enzyme itself can also be used (immobilized) inside the microreactor as well as within the solution. In one illustrative example, the synthesis of isoamylacetate using non-immobilized lipase in a microfluidic reactor showed superior performance with a 2.8 times faster reaction and 286% higher productivity [48]. Other similar systems have consistently shown both faster reaction times and higher productivity [47, 49, 50]. More complex oxygen-dependent reactions have also been performed in specialized microreactors supplying gas to the reaction surface [51].

**Fig. 4:**
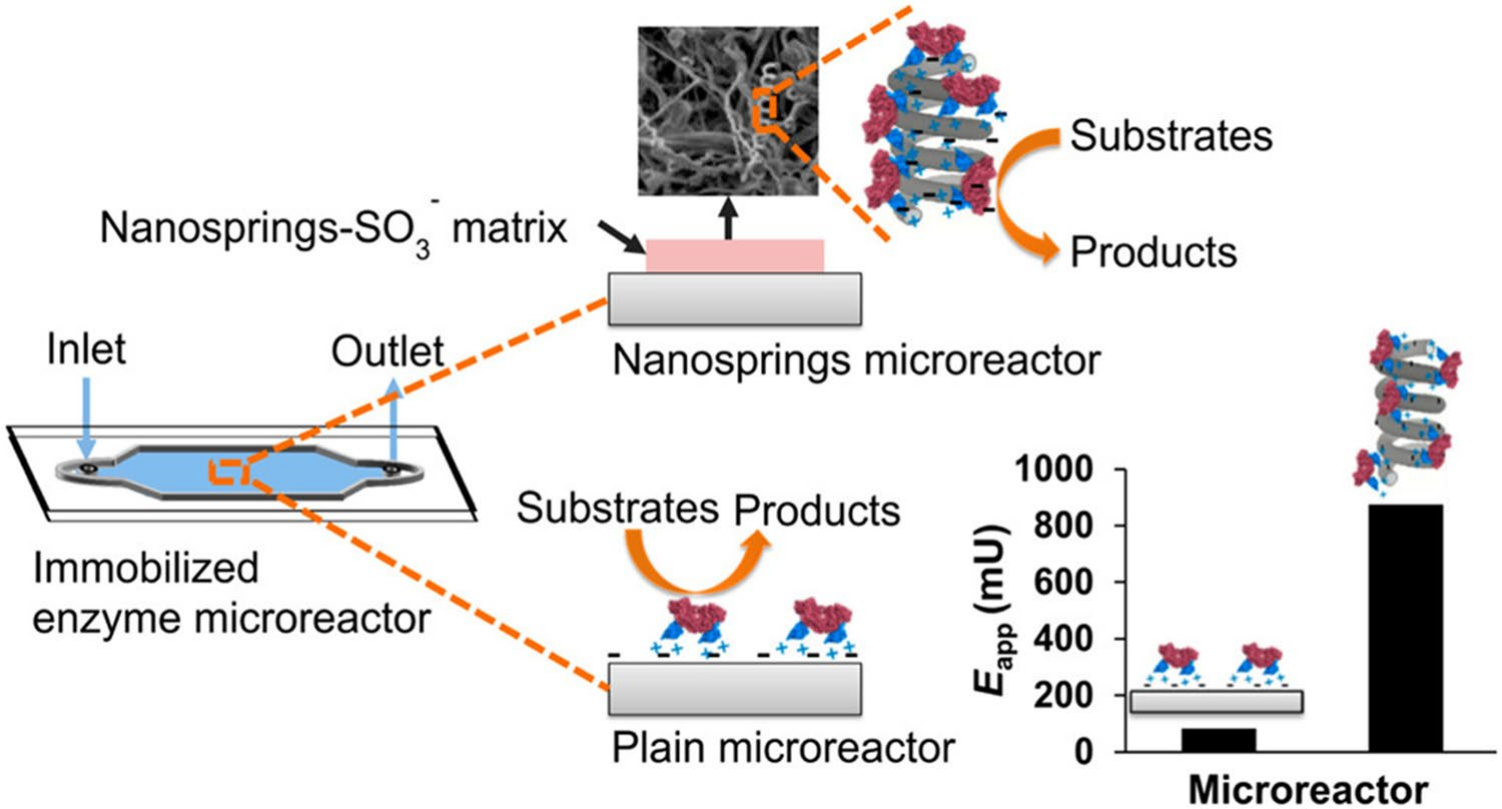
Immobilized enzyme microreactor with nanospring matrix to increase enzyme availability [54]. Reprinted with permission from Valikhani et al. Copyright 2017 American Chemical Society

Since the production of enzymes is often expensive, efficient and effective recovery of an enzyme *after* a reaction has been induced is usually highly desirable. Immobilizing an enzyme either inside the reactor or on larger particles is a popular method to enhance enzyme recover. Additionally, the stability of the enzyme can typically be improved via inducing immobilization [52]. Inappropriate enzyme immobilization can potentially lead to detrimental changes of the enzyme structure, however, which in turn lowers the enzyme activity. Microfluidic systems designed to immobilize enzymes can be classified into three types: the wall-coated type, the packed bed type, and the monolithic type [53]. In a wall-coated type microreactor, the enzyme is immobilized directly onto the channel walls. Although the surface to volume ratio is substantially higher in microfluidic systems when compared to conventional reactors, the wall size (and thus the enzyme loading capacity) remains relatively small, and the substrate diffusion path is comparably long. To mitigate these drawbacks, researchers have developed additional adhesion structures, like silica nanosprings, to increase the enzymatic adhesion layer (see Fig. 4) [54]. Alternatively, multiple layers of enzyme can also be loaded onto the channel wall, thereby increasing the availability of the enzyme in question [55]. By contrast, packed bed type microreactors fill the microfluidic channel with particles with immobilized enzyme. This leads to a great increase in enzyme loading capability when compared to wall-coated microreactors, along with a comparatively shorter diffusion path for the substrate [53]. For example, Kundu et al*.* used commercially available PMMA beads in a packed bed type reactor for continuous polymerization of polycaprolactone, and observed faster polymerization and higher molecular mass when compared to batch reactors [56]. Monolithic type microreactors aim to overcome the common issues of high pressure drops and the limited fluid flow and heat transfer (seen in densely packed bed type microfluidic channels) by filling the channel with interconnected porous structures. These have larger voids, which allow for easier fluid flow and lower pressure drop—which in turn generates higher productivity when compared to packed bed reactors [53]. Additionally, the encapsulation of enzymes inside matrices or membranes (while still allowing substrate and product movement) is another way of achieving enzyme immobilization which is applicable to microfluidic systems; for example, when Mizukami et al*.* used folded sheet mesoporous silicas to encapsulate lipases, and then compared a microreactor and a standard batch reactor, the microreactor exhibited a higher enzyme activity [57].

While enzyme screening using microfluidic technology has revolutionary improvements to throughput and speed, microfluidic enzyme bioreactors need to demonstrate tremendous benefits to compete with common bioreactor setups used in the industry. Additionally, lack of standardization and limitations in sensing technology for these miniaturized systems need to be overcome to implement microfluidic devices for enzyme bioprocesses [58].

### Identification and Analysis

#### Molecules

Detecting the presence of molecules within a given environment is a classic application for portable microfluidic systems. Biotechnology can aid in this type of task by using cells that are capable of biosensing various compounds—for example, certain types of microalgae can be used to detect pollutants such as herbicides, heavy metals, and volatile organic compounds [59]. In one experiment, a microfluidic dielectrophoresis system featuring the microalgae *Chlamydomonas reinhardtii* was used to detect mercury, methylmercury, copper, and copper nanoparticles via fluorescence detection [60]. In another microfluidic system, an algal fluorescence biosensor was deployed to detect the presence of the herbicide Diuron by using a light emitting diode and a photoreceptor that was integrated into the system itself [61]. And this approach is not limited to cells; it is also possible to use enzymes, antibodies, and even aptamers as biosensor recognition elements. These alternative biological components have been successfully used to detect small molecules, proteins, and even electrolytes and gases within microfluidic systems in the context of environmental analysis, clinical diagnostics, and food sample analysis [62].

#### Cells

Since biotechnology is defined as the use of biology to create useful products, it should not be surprising that there are cells involved in the majority of biotechnological applications. Identifying cells with the aim of discovering new cell lines is a key application with relevance across many fields of biotechnology—and the use of microfluidic systems for their cell identification functionalities can offer many advantages over more traditional lab-based instruments. Cell identification in microfluidic systems can be accomplished simply by using the optical, electrical, and/or biochemical properties of the cells, while simultaneously providing comparatively faster results (often at the point of sampling). This is a particularly critical advantage in marine biotechnology, where samples are drawn from waterways and oceans. Similarly, in food production, the rapid identification of bacterial or fungal contamination is a critical element for ensuring food safety; likewise in red biotechnology, fast detection and accurate identification of strains of bacteria and/or fungi is of the utmost importance for early diagnosis of a patient.

There are several different detection principles that have been successfully adapted for use within microfluidic devices. Microalgae can be detected using optical properties, such as light scattering or fluorescence. Electrical properties (like impedance and capacity) have also been successfully used for cell identification. For example, Benazzi et al*.* have developed a microfluidic cytometer that can be used to distinguish algal cells based on impedance measurements using two electrodes and fluorescence measurements. This device was able to detect algal cells larger than 2 µm, and distinguish between three different algae species that were tested [63]. Impedance measurements have also been deployed to detect *E. coli* contamination in food samples without the need for sample pre-enrichment or other preparatory treatments [64]. Similarly, the foodborne pathogen *Listeria monocytogenes* has been detected using impedance measurements and urease catalysis within a microfluidic system [65].

Another method for facilitating cell identification is via antigen detection. By using antibodies—which specifically bind to target cells—cells can be immobilized and detected. While this technique is broadly used in lateral flow assays for pregnancy tests and fast COVID detection, it is also applicable in microfluidic systems. This has tremendous applications in the sub-field of medical biotechnology, to the extent that it can help to provide point-of-care diagnostics for a fast diagnosis directly at the patient’s side. For example, Pham et al*.* have used a capillary driven microfluidic system to detect malaria antigens without the need for external pumps and at a sensitivity level that is suitable for early malaria detection [66].

More sensitive methods include detection of the RNA or DNA genome of the cell. In-situ hybridization can be used as a rapid detection technique, and the DNA or RNA probes used for detection are highly specific to the target. However, these types of assay protocols traditionally involve numerous loading, fixation, and washing steps—whereas microfluidic systems aim to simplify and streamline the entire operation. Fluorescence in situ hybridization (FISH) has already been demonstrated for both cancer prognosis [67–70] and species identification, and it can also be used with more complex tissue [67], food [71], or even environment samples [72].

The most famous method for genome analysis is undoubtedly polymerase chain reaction (PCR). PCR is a molecular biological method to replicate DNA using the enzyme polymerase, primers with a specific DNA sequence, and the sample DNA. This technique can be used to identify specific species in a complex sample, or even to identify specific mutations within the same species. PCR has been widely used in medical diagnosis [73], for food contaminant detection [74], for identification of microalgae [75] and in plant studies [76]. While early microfluidic PCR systems only performed the PCR itself on a microfluidic chip, modern solutions offer highly integrated systems that can include cell isolation, cell lysis, DNA extraction, PCR, and detection processes [77]. For example, Hong et al*.* have demonstrated an integrated PCR system for bacteria and mammalian cells which can even process multiple samples in parallel [78].

It should be noted that traditional PCR results represent the mean of a tested population—in other words, single mutant individuals cannot be detected within the larger population that is tested. By distributing the sample in a number of microscopic water droplets in an oil emulsion and performing the PCR reaction only *inside* of these droplets, however, a much more detailed analysis of variance can be performed. Since there is just a single cell contained in each droplet, every PCR result is essentially the analysis of a one cell population. This PCR method is called digital PCR [79], and the use of droplet microfluidics enables a significant increase in throughput from a few hundreds to *millions* of PCR results [80]. Pekin et al*.* have used a picolitre droplet microfluidic system to identify mutations in an oncogene with a sensitivity of detection one single mutation in 200,000 unmutated genes [81]. Similarly, Bian et al*.* have used this approach to detect pathogenic *E. coli* and *Listeria monocytogenes* in drinking water supplies [82].

**Fig. 5:**
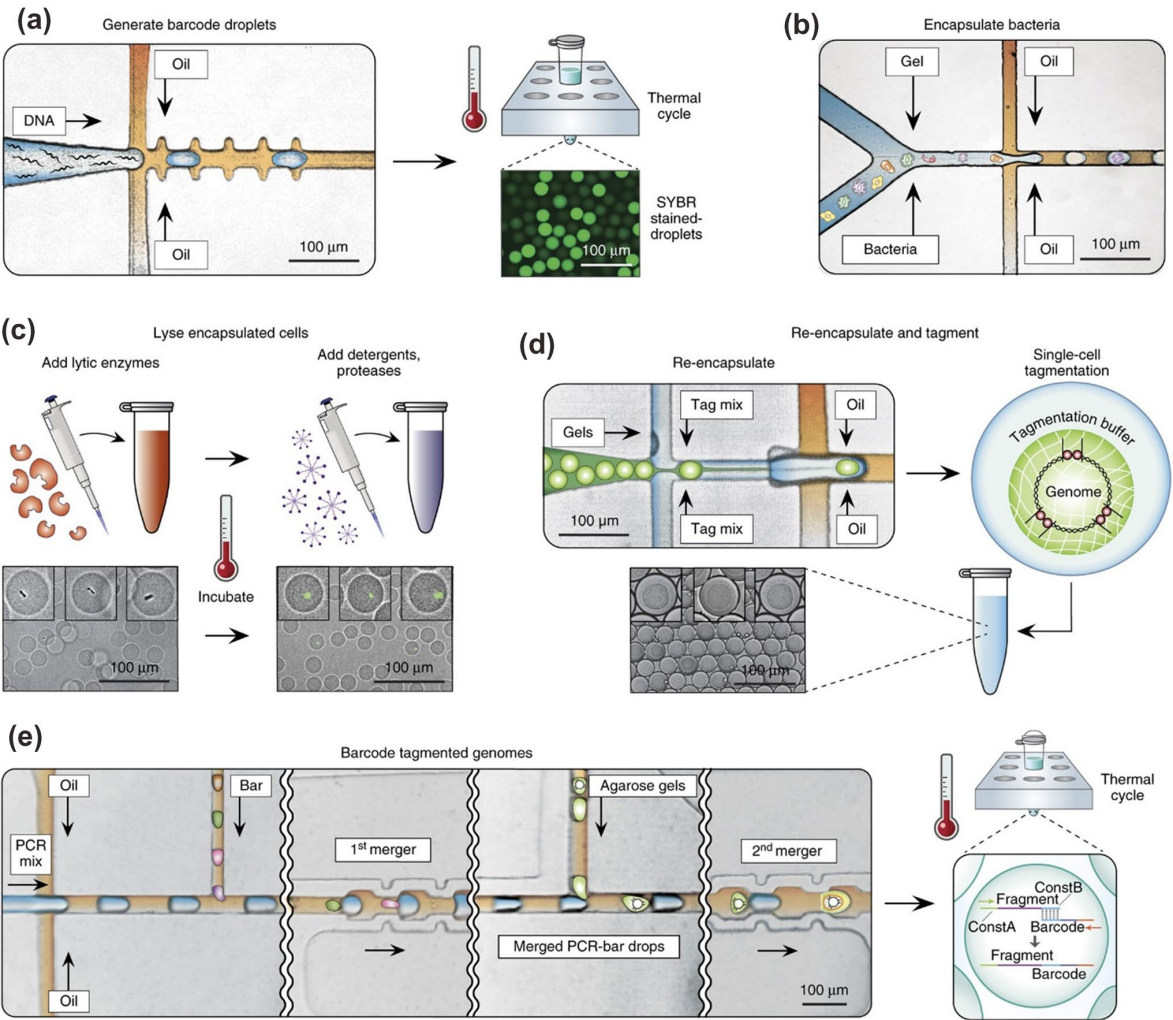
Droplet microfluidic system for single cell genome sequencing with droplet barcoding. **a** Generating barcode droplets by encapsulating random DNA oligomers and amplification by PCR. **b** Cells are encapsulated with molten agarose to generate microgels each containing a single cell. **c** The single cell genomes are purified through a series of bulk enzymatic and detergent lysis steps. **d** Microgels are re-encapsulated in droplets containing tagging (tagmentation) reagents. **e** The droplets containing tagmented genomes are merged sequentially with PCR reagents and barcode droplets, followed by PCR to splice barcodes to genomic fragments [88]. Reprinted by permission from: Nature Biotechnology Lan et al. (2017)

While PCR can be used to identify cells and detect mutations, DNA sequencing is required to actually identify each individual gene within the sample organism. The adaption of sequencing protocols to microfluidics [83] offers higher throughput and parallelization, as demonstrated by Aborn et al*.*, where the electrophoresis step of the Sanger method was parallelized up to 384 lanes. By using two microfluidic systems in parallel to enhance cleaning and loading steps, a remarkable 4 million bases per day was successfully sequenced [84]. Similar to single cell cultivation, microfluidics technology can also be used for single cell sequencing [85] to provide insights into the inherent heterogeneity of individual cells within a population, and can be used for both cancer [86] and immune disease detection [87]. Lan et al*.* have utilized a droplet microfluidic system for single cell sequencing by generating barcoded droplets, encapsulating bacteria in individual droplets, performing purification steps, and then merging barcode droplets and bacteria droplets to splice barcodes to genomic fragments (see Fig. 5) [88].

### Transferring Microfluidic Applications into Different Sub-Fields

Most of the microfluidic systems presented in this review originate from applications that have arisen in the sub-fields of red and white biotechnology. This can be attributed to the fact that these fields are more influential from an economical perspective, and therefore many model organisms and assays are deployed in research that occurs in these profitable areas. Other sub-fields of biotechnology also stand to benefit from the improvements made available by these systems by adapting these systems to their own specific needs, however. One example for this kind of knowledge transfer can be found by considering the relationship of organ-on-a-chip [89, 90] and plant-on-a-chip [91] devices. Both of these microfluidic applications offer similar benefits—i.e., the more accurate simulation of the physiological environment in which cells naturally exist, which allows researchers to better mimic real-world interactions between different cells and species and to analyze more closely their reaction to different stimuli like toxins. Of course, many other microfluidic technologies have also been successfully adapted across various sub-fields of biotechnology (see Chapter 2.3.2). Because microfluidic systems are frequently created using standard cell lines or simple assays for demonstrative purposes (which occur most often within the red and white biotechnology sub-fields), researchers working in other sub-fields stand to benefit tremendously from paying close attention to developments in those areas and then seeking to adapt new systems to their own specific research needs. Miniaturized analysis systems developed for use in food or marine biotechnology contexts could easily lead to faster and smaller analysis systems in a red or white biotechnology lab setting. Likewise, enzyme microreactor principles developed for white biotechnology could potentially find applicable in marine biotechnology analysis or production contexts.

## Conclusion

The utility of microfluidic systems has long been demonstrated for cell applications, enzyme applications, and identification/analysis applications across all biotechnology sub-fields. Because of their small size, these microfluidic systems require substantially less chemical or sample consumption when compared to traditional methods, while also simultaneously offering researchers new opportunities to exercise more precise reaction control over enzymes and physiological environments for cells. To date, however, it should be noted that most microfluidic systems have been developed in the red and white sub-fields of biotechnology—even though many other sub-fields also stand to benefit from a wider use of microfluidic systems. Part of this imbalance stems from the fact that, to a large degree, microfluidic systems still remain in the “demonstration” phase and have not yet achieved a tremendous degree of commercial application. Challenges remain in the lack of standardization and sensor miniaturization as well as the integration of microfluidic systems with pumps and analytic devices to create easy-to-use and reliable devices. As a result, most microfluidic systems created thus far have been developed around well-established applications in order to leverage specific benefits—which often involve the use of standard cell lines or assays from the sub-fields of red and white biotechnology. This only highlights the growing importance of cooperation between different research groups in order to facilitate the adaption of already published systems to other areas of biotechnology, however, since it is clear that the benefits of microfluidic systems can and should be expanded across all biotechnological sub-fields in a synergistic fashion.

## Data Availability

Not applicable.
